# Effect of Hydrocolloids and Emulsifiers on Baking Quality of Composite Cassava-Maize-Wheat Breads

**DOI:** 10.1155/2014/479630

**Published:** 2014-09-14

**Authors:** Maria Eduardo, Ulf Svanberg, Lilia Ahrné

**Affiliations:** ^1^Departamento de Engenharia Química, Faculdade de Engenharia, Universidade Eduardo Mondlane, Maputo, Mozambique; ^2^Department of Chemical and Biological Engineering/Food Science, Chalmers University of Technology, Gothenburg, Sweden; ^3^The Swedish Institute for Food and Biotechnology (SIK), Gothenburg, Sweden

## Abstract

Cassava is widely available worldwide but bread quality is impaired when cassava is used in the bread formulation. To overcome this problem, different improvers were tested in the preparation of composite cassava-maize-wheat (CMW) breads. Emulsifiers, diacetyl tartic acid ester of monoglycerides (DATEM), sodium stearoyl-2-lactylate (SSL), and lecithin (LC); and hydrocolloids, carboxymethylcellulose (CMC) and high-methylated pectin (HM pectin) were added during dough preparation of the composite flours (cassava-maize-wheat, 40 : 10 : 50). Each emulsifier was tested in combination with the hydrocolloids at levels of 0.1, 0.3, and 0.5% while hydrocolloids were used at a level of 3%. Bread quality attributes such as specific loaf volume, crust colour, crumb moisture, and firmness were measured. The specific volume of the fresh breads significantly improved with the addition of hydrocolloids (7.5 and 13%) and in combination with emulsifiers (from 7.9 to 27%) compared with bread produced without improvers. A significant improvement of brownness index and firmness of the composite flours breads was achieved with the addition of hydrocolloids and emulsifiers. The results show that emulsifiers and hydrocolloids can significantly improve the baking quality of CMW breads and thereby enhance the potential for using locally produced flours in bread baking.

## 1. Introduction

Use of composite flour in bread making has gained interest, especially in countries where wheat is not grown. According to FAO, 252 million tonnes of cassava was produced worldwide in 2011 [1]. This major food commodity has recently been promoted to be included in composite flour for bread making [2]. However, cassava contains no gluten, and partial substitution of wheat flour therefore impairs the quality of the bread. This effect has been attributed to reduced flour strength and gas retention capacity due to the lack of gluten proteins, thereby reducing bread volume and the sensory appeal of most baked composite bread [3]. To counteract these technological problems, several improvers have been used to mimic gluten properties.

Hydrocolloids are widely used to improve bread quality in wheat bread formulations [4, 5] and in gluten-free bread formulations in order to replace the viscoelastic and gas-binding properties of gluten [6–9]. Hydrocolloids also interact with the swelling, the gelatinization, and gelling properties of the dough and the retrogradation of the starch [10]. The type and dosage of hydrocolloids have significant effects on functional performance of the dough and subsequent bread quality. Xanthan gum has been reported to improve dough handling properties, loaf specific volume, and crumb softness when incorporated into composite cassava-wheat bread formulations [3]. Similar results were found by Yaseen et al. [11], who studied the use of gum arabic or pectin for the quality of corn-wheat pan bread. Correa et al. [12] showed that wheat bread with added high-methoxylated pectin (HM pectin) had a higher specific bread volume than bread with low-methoxylated pectin. The explanation may be the ability of HM pectin to establish hydrophobic interactions with gluten proteins through their methoxyl groups [13]. According to Collar et al. [5], some hydrocolloids will preferentially bind to gluten, for example, carboxymethylcellulose (CMC), or to the starch granules, for example, hydroxypropylmethylcellulose (HPMC). This interaction was associated with a significant displacement of endogenous gluten-bounded lipids to the starchy fraction (CMC) and with a significant decrease in lipids bound to the outside part of the starch granules (HPMC).

Emulsifiers are substances possessing both hydrophobic and hydrophilic properties, and various types are widely used in commercial bread formulas [14–16]. As emulsifiers include compounds with completely different chemical structures, they are therefore expected to have different effects on the dough and bread properties [17, 18]. Emulsifiers can function as dough strengtheners that mainly interact with gluten proteins and as crumb softeners or antifirming agents that can complex gelatinized starch [14, 19, 20]. The dough improving effect of emulsifiers seems to be related to their effect in reducing the repulsing charges between gluten proteins and thereby causing them to aggregate. This effect appears to be of particular importance in composite flours, as the wheat gluten has been diluted. Emulsifiers also contribute to retarding starch retrogradation by inhibiting the migration of water through interaction with starch molecules [4, 21, 22] and increasing gas cell stabilization in the dough by forming liquid lamellar films surrounding the gas cells [23, 24]. Sodium stearoyl-2-lactylate (SSL) and diacetyl tartaric acid esters of monodiglycerides (DATEM) are common anionic emulsifiers that have been shown to improve bread crumb and crust texture, and softness [4, 25] and to increase loaf volume [26, 27]. Due to their high hydrophilic/lipophilic balance, these improvers may promote aggregation of gluten proteins and form hydrogen bonds with glutamine and complex with starch granules [28] and thereby increase protein-starch interactions [29, 30]. These interactions generate a strong protein network and the development of a gluten-starch-lipid complex that as a result will produce bread with a better texture and increased volume [31, 32]. However, Gómez et al. [33] observed different effects on the gluten structure by SSL and DATEM. SSL with a higher hydrophilic/lipophilic balance (HLB) value will allow interaction with gluten proteins through ionic bonds while DATEM with its lower HLB mainly interacts with hydrophobic domains of gluten proteins [34]. Furthermore, DATEM was shown to have interactive effects on dough strength with HM pectin [35]. Ravi et al. [36] concluded that DATEM had greater effects on weak wheat flours than on stronger flours. Lecithin (LC) has been proven to increase specific volume in wheat bread [21] and to promote softer bread crumb compared with DATEM [26].

In gluten-free breads, a higher specific volume was obtained with SSL, DATEM, and LC that was related to the strength of the dough [37]. Indrani and Rao [38] reported the same trend for wheat bread.

Although the improving effects of emulsifiers and hydrocolloids in baking have been studied, there is scarce information about the combined effects of hydrocolloids and emulsifiers on the quality of composite breads containing cassava flours. The objective of this investigation was to improve the baking quality of composite bread in a study of the effects of an addition of two hydrocolloids (CMC and HM pectin) combined with different types of emulsifiers (DATEM, SSL, and LC) on specific volume, texture, colour, and moisture content of composite cassava-maize-wheat (CMW) bread.

## 2. Materials and Methods

### 2.1. Basic Ingredients and Improvers

Commercial wheat flour (Lilla Harrie Valskvarn AB, Göteborg, Sweden), yellow maize flour (AB Risenta, Sweden), dried active yeast (*Saccharomyces cerevisiae*), sodium chloride, sucrose, vegetable oil from soybeans, and ascorbic acid (GR, E. Merck) were used. Hydrocolloids were high-methylated pectin, HM pectin (GENU pectin type BIG, CP Kelco, Denmark), and carboxymethylcellulose, CMC (CEKOL 50000 W, CP Kelco, Denmark). Emulsifiers were diacetyl tartiric acid ester of monoglycerides, DATEM (MULTEC HP 20, Puratos, Belgium), sodium stearoyl lactylate, SSL (MULTEC 3000, Puratos, Belgium), and soy lecithin, LC (Sternchemie, Germany). All ingredients were purchased from commercial sources or directly from the suppliers. Fresh roots of cassava were obtained from local producers in Mozambique and then processed into flour of roasted cassava [2].

### 2.2. Methods

#### 2.2.1. Experimental Plan Design

A full factorial experimental design [39] without replicates was set up and 18 composite bread productions (3^2^∗2 = 18 sets without centre points) were carried out in random order (Table 1). The main experiment consisted of five factors, namely, the type of hydrocolloids (CMC and HM pectin) and type of emulsifiers (DATEM, SSL, and LC). Hydrocolloids were used at the same level of 3%, while the emulsifiers were added at three levels (0.1%, 0.3%, and 0.5%). The samples were analyzed for specific volume, moisture content, crumb firmness, and crust colour.

#### 2.2.2. Bread Processing

A bread recipe, based on flour weight, consisting of 500 g of flour (roasted cassava 40%, maize 10%, and wheat 50%), 1.6% dry yeast, 1.5% salt, 3% vegetable oil from soybeans, 0.1% ascorbic acid, and 88.3% water, was used in this study. The bread processing followed a planned design presented in Table 1. The baking procedure and conditions were the same as in the previous study [2] and were as follows. The ingredients were mixed for 10 min in a mixer (Kitchen Aid, KSM9, Michigan, USA), allowed to rest at room temperature for 45 min, divided into 20 loaves (50 g each), hand molded, and placed into bread pans. Dough was proofed at 30°C and 80% relative humidity for 45 minutes. The loaves were baked at 220°C for 7 min in a convection oven (Dahlen S400, Sveba Dahlen AB, Sweden). After baking, the loaves were removed from the pans and allowed to rest for cooling for 60 min at ambient conditions (25°C, 50%) before weighing. A composite cassava-maize-wheat (CMW) bread sample with no improvers was used as a control. Cooled bread samples were packaged in polypropylene bags to prevent moisture loss and were used for further analysis.

### 2.3. Technological Evaluation of the Bread

#### 2.3.1. Specific Loaf Volume Measurement

Loaf volume was determined using the rapeseed displacement method, but using alfalfa seeds instead of rapeseeds. Each loaf (*n* = 6) was weighed and the volume was measured 60 min after being taken from the oven. The specific loaf volume was reported as cm^3^/g of the loaf.

#### 2.3.2. Crust Colour

The colour was measured 180 min after baking in four loaves. Crust colour was quantified with the Digital Colour Imaging System (DigiEye) (Cromocol Scandinavia AB, Borås, Sweden). The controlled illumination cabinet on the DigiEye equipment was utilized to capture high resolution images of the fresh bread surface. The DigiEye 2.53b software (Cromocol Scandinavia AB, Borås, Sweden) allows for storage of specific colour standards with a given *L* ^*^ (lightness), *a* ^*^ (redness-greenness), and *b* ^*^ (yellowness-blueness) values according to the CIELAB definition. The results were reported as brownness index (BI), calculated according to Maskan [40]:

(1) $$\begin{array}{c} \text{BI}=\frac{\left[100\left(x-0.31\right)\right]}{0.17}, \end{array}$$

where

(2) $$\begin{array}{c} x=\frac{a+1.75L}{5.645L+a-3.01b}. \end{array}$$

#### 2.3.3. Crumb Firmness

The crumb firmness was measured 6 h after baking using an Instron Universal Testing Machine (UTM, model 5542). The AACC standard method 74-09 was used. The measurements were carried out on 25 mm-thick slices taken from the centre part of the loaf of bread. Samples were compressed to approximately 10 mm (40% of the thickness of the slice) at a test speed of 1.7 mm/s. The measurements were carried out on four loaves from each batch, and the compression force (in Newton) at the end of the compression was defined as firmness.

#### 2.3.4. Moisture Content

The moisture content of fresh bread samples (*n* = 3) was determined by drying overnight in a vacuum oven at 70°C [41].

#### 2.3.5. Crumb Grain Structure Analysis

The grain structure of the crumb was analyzed using pictures of composite bread crumbs taken with the Digital Colour Imaging System (DigiEye) (Cromocol Scandinavia AB, Borås, Sweden) and Matlab software [42]. Images were stored in a TIF format of 4288 ∗ 2848 pixels, and a region with approximately 900 ∗ 900 pixels was selected. The parameter measured was the mean cell area, expressed in mm^2^. This is the average of all cells' area:

(3) $$\begin{array}{c} \text{Mean}\;\text{cell}\;\text{area}\left(\text{MCA}\right)=\frac{\text{Total}\;\text{area}\;\text{of}\;\text{cells}}{\text{Total}\;\text{number}\;\text{of}\;\text{cells}}. \end{array}$$

This characteristic gives an idea of the size of the cells of the bread crumb.

### 2.4. Statistical Analysis

All experiments were performed in a completely randomized design. A statistical difference in bread properties was determined by one-way analysis of variance (ANOVA) and Tukey's HSD post hoc multiple range test (*P* < 0.05).

## 3. Results and Discussion

Tables 2 and 3 show the results for specific loaf volume, moisture content, firmness, and brownness index value of the composite cassava-maize-wheat (CMW) bread loaves prepared without emulsifiers and hydrocolloids and prepared with the combinations of CMC or HM pectin as hydrocolloids and DATEM, SSL, or LC as emulsifiers.

There were significant (*P* < 0.05) differences in specific volume, firmness, and brownness index values between CMW control breads without improvers and bread samples with hydrocolloids and emulsifiers. The effect of these baking improvers on quality parameters was dependent on the type of hydrocolloid and level of emulsifier. An increase was observed for specific volume and crust colour values while crumb firmness showed a decrease. Crumb moisture content did not change as an effect of adding emulsifiers and was in the range from 47.3 to 48.6% (w/w).

### 3.1. Specific Volume of the Loaves

The specific volume of the breads increased (*P* < 0.05) as an effect of added hydrocolloids at a level of 3%, from 1.94 cm^3^/g in the CMW control bread to 2.14 cm^3^/g with CMC and to 2.07 cm^3^/g with HM pectin; see Tables 2 and 3. Similar effects on specific bread volume have been reported with additions of xanthan gum, HPMC, and *κ*-carrageenan to wheat bread [8], of HPMC to gluten-free maize-teff bread [43], and of pectin and CMC to gluten-free formulations [44]. These findings might be a result of the formation of a gel network during oven heating that strengthens the expanding cells of the dough and, as a result, improves gas retention and bread volume [45]. Roasted cassava flour with pregelatinized starch granules and partly leached amylose may also contribute to build up a network with the hydrocolloids during the dough phase [46]. A network of this kind with pregelatinized cassava could improve gas-holding properties in the dough phase [47] and has been shown to result in a higher specific volume of sorghum-cassava bread [48, 49].

**Figure 1:**
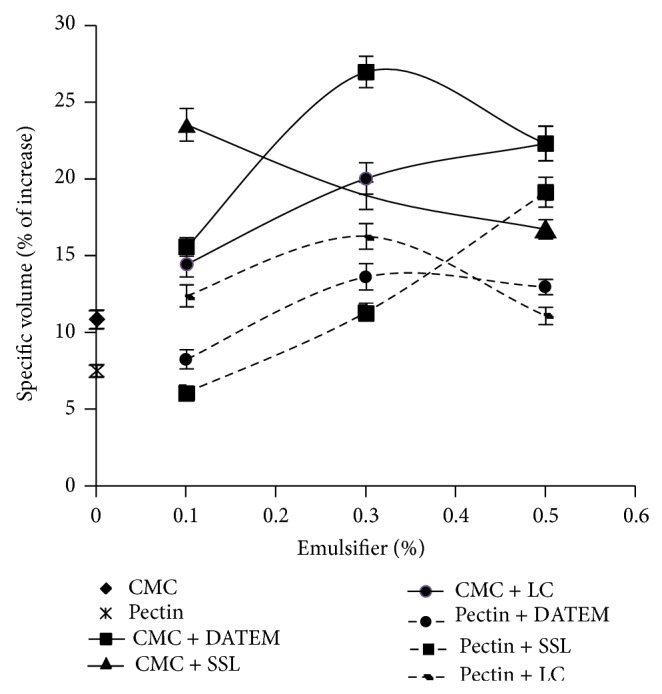
Specific volume increase (%) due to hydrocolloids CMC and pectin at a level of 3% and emulsifier types (DATEM, SSL, and LC) at different levels of addition. The error bars represent the standard deviation.

Compared with control bread, the specific volume increased further (*P* < 0.05) with the addition of emulsifiers, depending on the type and concentration; see Figure 1. For breads with CMC, the specific volume increased between 23 and 27% to 2.46 cm^3^/g with DATEM (0.3%), 2.40 cm^3^/g with SSL (0.1%), and 2.38 cm^3^/g with LC (0.5%). In breads with HM pectin, the increase in the specific volume was comparatively lower, between 14 and 19%, with the highest value, 2.31 cm^3^/g, in bread with SSL at a level of 0.5%. The larger specific volume of CMW bread loaves prepared with CMC compared with HM pectin may be explained by a higher ability of CMC to interact with the gluten proteins, resulting in a more stable dough and better volume [5, 12].

Rogers and Hoseney [50] reported that additions of DATEM and SSL improved the loaf volume of wheat bread. Similar results were reported by Nunes et al. [37] when LC and DATEM (0.5%) were added in the formulation of gluten-free bread formulations. The positive effect on bread volume of emulsifiers is caused by their dough strengthening properties by forming liquid films with a lamellar structure in the interphase between the gluten strands and the starch [14]. Krog [51] reported that SSL and DATEM were the most effective in creating this lamellar structure.

Our results can be explained on the basis of the structural properties of the emulsifiers. The amphiphilic nature of emulsifiers may contribute to the strength of the gluten network; in general, anionic emulsifiers, such as DATEM and SSL, will increase the strength of the dough by interacting with hydrophobic regions of the gluten proteins and forming hydrogen bonds with the amino groups of glutamine [14, 19, 26, 52]. The improved dough strength will allow better carbon dioxide retention during oven spring and, as a result, will give bread with improved loaf volume [26, 53]. However, an addition of lecithin has been shown to reduce the stability of wheat flour dough [19].

### 3.2. Crumb Moisture

The moisture content in the crumbs of breads increased slightly with added hydrocolloids (47.9-48.0%) in comparison with the CMW control bread (47.2%). These results are in agreement with the work of Bárcenas and Rosell [54], who reported that the addition of HPMC increased the crumb moisture content of white bread. In general, crumb moisture was unaffected by the addition of an emulsifier.

### 3.3. Crumb Firmness

Data on texture of breads with different emulsifiers are presented in Tables 2 and 3.

Hydrocolloids reduced the crumb firmness compared to the CMW bread (7.1 ± 0.25 N). The reduction in the crumb firmness with the addition of either CMC or HM pectin was about 34% and 14%, respectively. According to Biliaderis et al. [55], hydrocolloids may decrease granular swelling of the starch and the amount of amylose leached from the granules and, as a result, hinder a building up of an amylose network and thereby result in a softer crumb. On the other hand, the roasted cassava flour used in the composite flour mixture is highly gelatinized with partially swelled starch granules and retrograded leached amylose [46]. The linear polysaccharide in CMC may interfere with the amylose of the gelatinized cassava starch granules breaking the gel network, which might explain the softening of the bread crumb [56]. The better effect of CMC compared with HM pectin is in line with a relatively higher volume of CMC bread, which might be explained by a better affinity of CMC to gluten than that of HM pectin [5].

Emulsifiers that can complex amylose are more efficient in reducing bread firmness than those that do not form complexes. The higher the complex-forming power of the emulsifier, the lower the initial bread crumb firmness [14, 57]. Monoacylglycerols are known as the most efficient crumb softeners, and both SSL and DATEM, with one stearic acid in the structure, might be expected to have a similar effect on bread firmness. In bread with CMC, the addition of emulsifiers had a lower effect on crumb firmness as compared with HM pectin bread. The highest reduction was observed for SSL at the level of 0.1% (18.3%), followed by LC (9.1%) and DATEM (6.8%), both at the level of 0.3% (*P* < 0.05).

In HM pectin bread, however, the addition of emulsifiers had stronger crumb softening effects, and the crumb firmness with LC was 4.71 N, which was 35% lower as compared with CMW bread. The reducing effect of DATEM and SSL on firmness has been reported for wheat breads by Ribotta et al. [27]. Gómez et al. [26] reported the same effect by lecithin. The positive effect of DATEM has been explained by its capacity to aggregate gluten proteins, which create a gluten network that can improve the entrapment of air and result in better bread volume and crumb texture.

**Figure 2:**
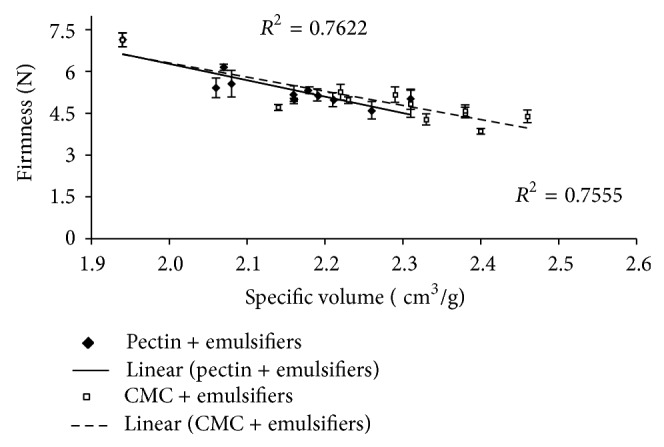
Correlation between firmness of the composite bread crumb and the specific volume. The error bars represent the standard deviation.

Figure 2 shows similar correlations between firmness and specific volume of the composite breads with CMC (*R* ^2^ = 0.762) and HM pectin (*R* ^2^ = 0.755) and, as expected, these two properties are strongly correlated and do not significantly depend on the type of emulsifier. Higher values in the specific volume led to a softer bread crumb. However, the lower value of the correlation coefficient may indicate that the volume change is not the only factor causing reduction of bread firmness.

### 3.4. Crust Colour

The brownness index of bread samples with either CMC or HM pectin was increased by 40% and 16%, respectively, compared with CMW bread (BI value of 53.3 ± 1.9).

The literature gives conflicting results on crust colour as an effect of hydrocolloids. Lighter bread was obtained with xanthan gum in composite cassava-wheat bread [3]; darker bread was obtained in gluten-free formulations with xanthan gum [58], HPMC, and carrageenan [9]; lighter bread resulted with CMC [58] and xanthan gum [9]; and no effect on crust colour was observed with pectin, CMC, and xanthan [44].

With the addition of emulsifiers, the BI values significantly increased in all breads containing HM pectin. With DATEM, the BI values increased from 66 to between 82 and 98, with the highest value at the 0.3% level. The BI values in loaves with SSL varied between 77 and 85 and in loaves with LC between 74 and 80. However, there was no effect on the BI values with addition of SSL to the CMC bread (BI value of 80), while the BI values significantly increased with an addition of DATEM (up to 95) and an addition of LC (up to 87).

Thus, several composite breads with hydrocolloids and emulsifiers had BI values in the range from 82 to 86, values that received a high score in a consumer acceptance test of CMW breads [59].

### 3.5. Crumb Grain Structure

**Figure 3:**
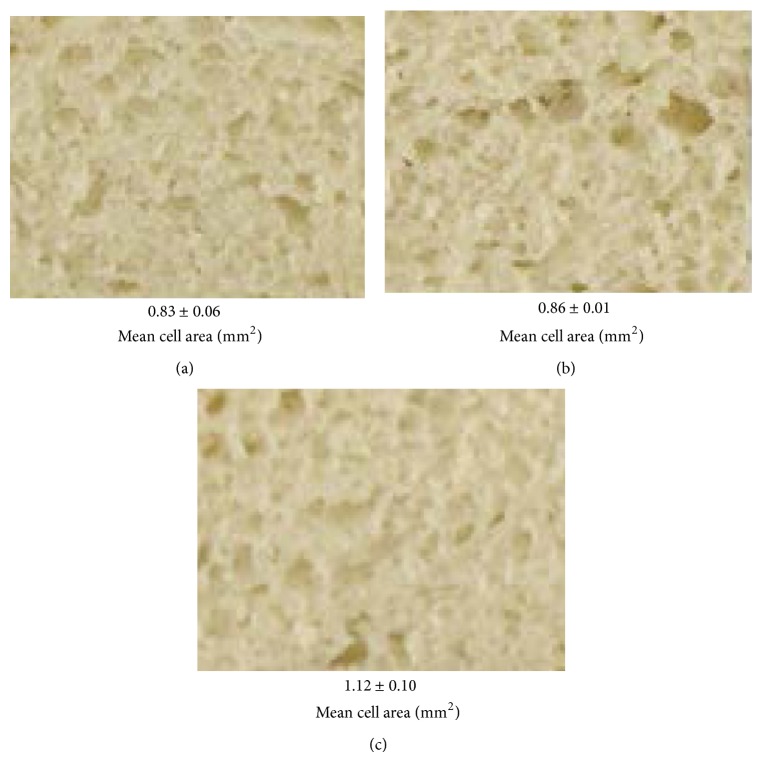
Crumb grain structure showing the mean cell area (mm^2^) for composite cassava-maize-wheat bread (a) without improvers; (b) +CMC; and (c) +HM pectin.

The grain structure of the crumb in CMW breads as it is affected by the hydrocolloids and emulsifiers has been described by image analysis using the mean cell area (mm^2^) (Table 4). Bread baked with HM pectin showed a higher mean cell area (1.12 ± 0.10 mm^2^) compared with CMC (0.86 ± 0.01 mm^2^) and composite cassava bread (0.83 ± 0.10 mm^2^) without any improver, which means a more porous crumb structure (Figure 3). However, an addition of SSL to loaves containing pectin significantly reduced the mean cell area to between 0.76 and 0.83 mm^2^. On the other hand, in composite bread with CMC, the mean cell area was significantly increased by an addition of emulsifiers at 0.3% level. These results are in accordance with a higher specific volume that was observed for these breads (Table 3).

## 4. Conclusions

Addition of either CMC or HM pectin as baking improvers to composite cassava-maize-wheat (CMW) bread loaves improved bread quality parameters such as specific volume, crust colour, and crumb texture. In general, emulsifiers in combination with CMC had a more positive effect on bread specific volume compared with HM pectin, which can be explained by differences in interactions of CMC or pectin with emulsifiers and the gluten protein network created in the composite bread. The crumb grain structure was greatly affected by pectin or by a combination of CMC and emulsifiers (especially DATEM and LC).

Overall, the results of this study suggest that the quality of CMW breads can be significantly improved by an addition of hydrocolloids or mixtures of hydrocolloids and emulsifiers as baking improvers. These baking improvers enhance the potential of using locally produced flours, such as cassava and maize, in composite flour for bread making in South East Africa.

## Figures and Tables

**Table 1:** Levels and types of emulsifiers and hydrocolloids added to composite cassava-maize-wheat (CMW) bread formulations.

Experiment number	Emulsifier	Level of emulsifier (% w/w)	Hydrocolloid (3% w/w)
1	DATEM	0.1	CMC
2	DATEM	0.1	HM pectin
3	SSL	0.1	CMC
4	SSL	0.1	HM pectin
5	LC	0.1	CMC
6	LC	0.1	HM pectin
7	DATEM	0.3	CMC
8	DATEM	0.3	HM pectin
9	SSL	0.3	CMC
10	SSL	0.3	HM pectin
11	LC	0.3	CMC
12	LC	0.3	HM pectin
13	DATEM	0.5	CMC
14	DATEM	0.5	HM pectin
15	SSL	0.5	CMC
16	SSL	0.5	HM pectin
17	LC	0.5	CMC
18	LC	0.5	HM pectin

**Table 2:** Specific loaf volume, moisture content, firmness, and brownness index value of composite cassava-maize-wheat (CMW) breads without hydrocolloids or emulsifiers.

Composite bread	Specific volume (cm^3^/g)	Moisture content (%)	Firmness (N)	Brownness index
CMW without hydrocolloids or emulsifiers	1.94 ± 0.06	47.2 ± 0.5	7.14 ± 0.3	57.3 ± 1.9

**Table 3:** Effect of type and concentration of hydrocolloids^1^ and emulsifiers^2^ on specific loaf volume, moisture content, firmness, and brownness index value in composite cassava-maize-wheat (CMW) breads.

Composite bread	Level of emulsifier (%)	Specific volume (cm^3^/g)		Moisture content (%)		Firmness (N)		Brownness index	
		CMC	HM-pectin	CMC	HM-pectin	CMC	HM-pectin	CMC	HM-pectin
No emulsifier	0	2.14^a^	2.07^bc^	47.9 ± 0.1	48.0 ± 0.1	4.71^de^	6.15^e^	80.0^ab^	66.2^a^

+DATEM	0.1	2.23^ab^	2.08^bcd^	47.5 ± 0.4	48.6 ± 1.7	4.97^ef^	5.56^d^	88.8^d^	82.3^de^
	0.3	2.46^e^	2.21^de^			4.39^bc^	4.99^b^	87.9^cd^	97.9^g^
	0.5	2.38^cde^	2.19^de^			4.59^cd^	5.16^bc^	95.2^e^	93.2^f^

+SSL	0.1	2.40^de^	2.06^ab^	47.3 ± 0.3	47.5 ± 0.5	3.85^a^	5.41^cd^	84.1^bc^	84.7^e^
	0.3	2.31^bcd^	2.16^bcde^			4.84^de^	4.98^b^	84.8^cd^	76.9^bc^
	0.5	2.29^bc^	2.31^f^			5.17^f^	5.00^b^	76.7^a^	85.3^e^

+LC	0.1	2.22^ab^	2.18^cde^	47.4 ± 0.3	47.6 ± 0.3	5.26^f^	5.33^bcd^	87.0^cd^	78.4^cd^
	0.3	2.33^cd^	2.26^de^			4.28^b^	4.61^a^	86.1^cd^	79.9^cd^
	0.5	2.38^cde^	2.16^bcde^			4.53^bcd^	5.16^bc^	85.1^cd^	73.8^b^

^1^CMC: cellulose gum, HM pectin: high methoxyl pectin at 3% (w/w) level, ^2^DATEM: diacetyl tartaric acid ester of mono- and diglycerides, SSL: sodium stearoyl-2-lactylate, and LC: soy lecithin (% in w/w).

^a,b,c,d,e,f,g^Different letters in the same column represent values that are significantly different (*P* < 0.05).

**Table 4:** Mean cell area of composite cassava-maize-wheat (CMW) bread as affected by the hydrocolloids (CMC and HM pectin) and emulsifier types (DATEM, SSL, and LC) at different levels of addition.

Composite bread	Level of emulsifier (%)	Mean cell area (mm^2^)∗	
		CMC	HM-pectin
No emulsifier	0	0.86 ± 0.01	1.12 ± 0.10

+DATEM	0.1	1.18 ± 0.21	1.23 ± 0.10
	0.3	1.39 ± 0.25	1.00 ± 0.12
	0.5	0.96 ± 0.03	1.08 ± 0.02

+SSL	0.1	0.74 ± 0.01	0.81 ± 0.15
	0.3	0.94 ± 0.03	0.76 ± 0.03
	0.5	0.80 ± 0.05	0.83 ± 0.00

+LC	0.1	0.75 ± 0.15	1.17 ± 0.01
	0.3	1.54 ± 0.07	0.92 ± 0.04
	0.5	0.89 ± 0.07	0.88 ± 0.12

^*^The values are represented by mean value (*n* = 2)  ± standard deviation.
